# Genetic predisposition, parity, age at first childbirth and risk for breast cancer

**DOI:** 10.1186/1756-0500-5-414

**Published:** 2012-08-07

**Authors:** Salma Butt, Sophia Harlid, Signe Borgquist, Malin Ivarsson, Göran Landberg, Joakim Dillner, Joyce Carlson, Jonas Manjer

**Affiliations:** 1 Department of Surgery, Skåne University Hospital, Lund University, Malmö, Sweden; 2 Department of Medical Microbiology, Skåne University Hospital, Malmö, Sweden; 3 Department of Clinical Chemistry, Skåne University Hospital, Malmö, Sweden; 4 Department of Oncology, Skåne University Hospital, Lund, Sweden; 5 Breakthrough Breast Cancer Research Unit, Paterson Institute for Cancer Research, Manchester University, Manchester, UK; 6 Departments of Laboratory Medicine, Medical Epidemiology & Biostatistics, Karolinska Institute, Karolinska University Hospital, Stockholm, Sweden; 7 Department of Laboratory Medicine, Skåne University Hospital Lund, Lund, Sweden; 8 Department of Plastic and Reconstructive Surgery, Skåne University Hospital, Lund University, Malmö, Sweden; 9 The Malmö Diet and Cancer Study, Skåne University Hospital, Malmö, Sweden

## Abstract

**Background:**

Recent studies have identified several single-nucleotide polymorphisms (SNPs) associated with the risk of breast cancer and parity and age at first childbirth are well established and important risk factors for breast cancer. The aim of the present study was to examine the interaction between these environmental factors and genetic variants on breast cancer risk.

**Methods:**

The Malmö Diet and Cancer Study (MDCS) included 17 035 female participants, from which 728 incident breast cancer cases were matched to 1448 controls. The associations between 14 SNPs and breast cancer risk were investigated in different strata of parity and age at first childbirth. A logistic regression analysis for the per allele risk, adjusted for potential confounders yielded odds ratios (OR) with 95% confidence intervals (CI).

**Results:**

Six of the previously identified SNPs showed a statistically significant association with breast cancer risk: rs2981582 (FGFR2), rs3803662 (TNRC9), rs12443621 (TNRC9), rs889312 (MAP3K1), rs3817198 (LSP1) and rs2107425 (H19). We could not find any statistically significant interaction between the effects of tested SNPs and parity/age at first childbirth on breast cancer risk after adjusting for multiple comparisons.

**Conclusions:**

The results of this study are in agreement with previous studies of null interactions between tested SNPs and parity/age at first childbirth with regard to breast cancer risk.

## Background

The risk of breast cancer among first-degree relatives of a breast cancer patient is about twice as high as in the general population [1]. The genes BRCA1 and BRCA2 are associated with the risk of breast cancer [2], however these genes account for only 30-40% of the familial breast cancer cases, and only 3-4% of the total number of breast cancer cases [2]. A much larger proportion of all cases have been associated with environmental factors such as reproductive history, life-style and endogenous hormonal levels. Two important factors associated with breast cancer risk are parity and age at first childbirth [3].

Genome wide association studies (GWAS) have recently identified several single nucleotide polymorphisms (SNPs) associated with breast cancer risk [4-7]. Certain combinations of these polymorphisms and reproductive factors could affect the susceptibility for breast cancer.

The Malmö Diet and Cancer Study (MDCS) is a prospective population-based cohort in Malmö, Sweden. It provides tumour endpoints, DNA samples, and information on parity and other reproductive factors for a total of 17 035 women. The aim of the present nested-case control study was to study whether the reported associations between reported risk SNPs and breast cancer differ by parity and age at first birth.

## Methods

### The Malmö Diet and Cancer Study (The MDCS)

The MDCS, a population-based prospective cohort study recruited participants between 1991 and 1996. All female residents of Malmö, Sweden, born between 1923 and 1950 were invited. Written informed consent was obtained by all participants at baseline. In all, 41% of invited women participated, and the female cohort consisted of 17 035 women [8]. Baseline examinations included a questionnaire providing information on parity, age at first childbirth, education, occupation, marital status, age at menarche, age at menopause, exposure to oral contraceptives (OC) (ever/never), current use of hormonal replacement therapy (HRT), alcohol consumption and smoking habits [9]. HRT was defined as non-use, use of estrogen replacement therapy, progesterone replacement therapy or combined hormonal replacement therapy. Information on gynecological surgery was collected from medical records and menopausal status was defined, using this information together with data obtained from the questionnaire on menstruations, as previously described in detail [10]. A trained nurse at the study centre measured height and weight, and Body Mass Index (BMI) was calculated as kg/m^2^[9].

The MDCS and the present analyses were approved by the Ethical Committee at Lund University (LU 51–90, Dnr 652/2005 and Dnr 2009/682).

### Parity and age at first childbirth

Information on parity was assessed from the questionnaire. The question: “How many children have you given birth to and in what years were they born?” was categorised as; nullipara, one child, two children and three or more children. Parity was further dichotomized as nulliparous and parous in order to yield larger groups. Age at first childbirth was calculated from the information provided in the same question and categorised as; ≤20, >20 - ≤25, >25 - ≤30 and >30. Age at first childbirth was also dichotomized as ≤ 25 years of age and > 25 years of age. Information on parity and age at first childbirth was missing for a small number of women, and they were excluded from all analyses. No reliable information on twin pregnancies was available nor on miscarriages or abortions.

### Follow-up

All women were followed until 31st of December 2007. Tumour end-points were retrieved by record linkage with The Swedish Cancer Registry (until 31st of December 2006), and due to a delay in central registration, also with linkage to its regional branch, The Southern Swedish Regional Tumour Registry concerning 2007. Vital status was obtained from The Swedish Cause-of-Death Registry until 31st of December 2007.

### Study population

All 17035 women were followed for cancer as described above. For the purpose of the present study, only women with no previous (prevalent) cancer (not including cancer in situ of the uterine cervix) were eligible for inclusion. A total of 545 cases with incident breast cancer were identified in a first set with follow-up until 31st of December 2004. One case did not have any DNA sample hence this case was excluded. The remaining 544 cases were matched to two controls each, a total of 1088. The matching criteria were age (+/− 90 days) and time of sampling at baseline (+/− 30 days). A new linkage was performed with follow-up until 31st of December 2007, where an additional 186 cases and 372 controls were identified. A total of 11 controls from the first set were diagnoses with breast cancer during the second follow-up period. They were removed as controls and replaced by other controls matched on the same criteria. For 14 women, there were no DNA available for sampling (2 cases and 12 controls); hence they were excluded from all statistical analyses. Finally, the study population consisted of a total of 2176 women out of which 728 were cases and 1448 were controls.

### SNP selection and analysis

Eleven SNPs were selected from previous GWAS and candidate SNP publications by Easton et al. (rs2981582 (FGFR2), rs3803662 (TNRC9), rs12443621 (TNRC9), rs98051542 (TNRC), rs889312 (MAP3K1), rs3817198 (LSP1), rs2107425 (H19), rs13281615 (8q24), rs981782 (5p12), rs30099 (5q), rs4666451 (2p)) [4], one from Cox et al. (rs1045485 (CASP8)) [7], one from Stacey et al. (rs13387042 (2q35)) [5] and one from Harlid et al. (rs7766585 (ESR1)) [11]. The GWAS SNPs were chosen from studies published up until the 1st of July 2007.

The tested SNPs were analysed in a previous larger data set including other centres. For the current cohort, a screening and a verification test was performed in 1605 individuals. The comparison showed concordant results in 99% of analyses [11].

The SNP analyses were performed with a MALDI-TOF mass spectrometer (SEQUENOM MassArray) using iPLEX reagents and protocol (SEQUENOM) and 10 ng DNA as PCR template. Primer sets were from Metabion (Martinsried, Germany). The laboratory methods have previously been described in detail [11].

The genotypes for the SNPs were defined as: homozygous major allele (AA), heterozygote (Aa) and homozygous minor allele (aa). In cases with minor allele frequency (MAF) near 0.5, the same classification as that used in previous studies was used.

### Statistical methods

Cases and controls were compared with regard to established and potential risk factors for breast cancer in order to identify possible confounders.

An unconditional binary logistic regression model was fitted to analyse the association between SNPs and breast cancer. A per allele analysis was performed using a continuous variable with the values 0 (AA), 1 (Aa), and 2 (aa). The reported odds ratios (OR) with 95% confidence intervals (CI) denotes the risk difference when increasing the number or risk alleles with one. In addition ORs were calculated using the common allele (AA) as reference group for risk estimates for the separate genotypes in all analyses.

Parity and age at first birth was dichotomised as nulliparous vs. parous and ≤ 25 years of age at first childbirth vs. age > 25 years of age at first childbirth. Overall breast cancer risk was calculated with the lowest groups as reference, adjusted for birth year and year at baseline.

The SNP analyses were stratified on nulliparous vs. parous women and on age ≤ 25 years of age at first childbirth vs. age > 25 years of age at first childbirth. Parity and age at first childbirth were also studied in four categories.

In addition, the material was stratified on single genotypes, and the breast cancer risk associated with parity and increasing age at first birth was calculated. These associations were reported using the p-values for the continuous analysis.

All analyses were subsequently adjusted for matching criteria, age and year of inclusion in study, and for potential confounders. A potential confounder was defined as a factor with a distribution difference exceeding 5% units between cases and controls and only these factors were included in the multivariate analysis.

In order to assess any potential interactions between selected SNPs and parity and between selected SNPs and age at first birth, an interaction-term was introduced in the logistic regression model. A p-value < 0.05 was considered statistically significant. In a third step, the p-value was corrected for multiple comparisons according to Bonferroni, i.e. divided by the number of comparison. In the present study, performing 28 interaction analyses, the corrected p-value regarded as statistically significant was 0.0018.

As part of a sensitivity analysis, all analyses were repeated excluding women with information on less than 80% of SNPs in a single individual, as this may indicate a poor DNA quality. In these analyses 660 cases and 1310 controls were included.

## Results

### Case–control status and distribution of potential confounders

Among the described variables, cases were more often non-manual workers as compared to controls; 60% vs. 52% (Table 1). More cases were users of HRT, particularly combined hormonal replacement therapy (CHRT), as compared to controls 21% vs. 12% (Table 1). These factors differed by at least 5% units between cases and controls and were, hence, included in the multivariate analyses. All other factors were similarly distributed between cases and controls.

**Table 1:** Case–control status and distribution of potential confounders

**Factor**	**Category**	**Case (n = 728)**	**Control (n = 1448)**
		**Column% (n) *Mean (SD) in italics* **	
Education	O-level college	67.9 (494)	70.2 (1017)
	A-level college	7.1 (52)	7.0 (101)
	University	24.7 (180)	22.4 (325)
	Missing	0.3 (2)	0.3 (5)
Type of occupation	Manual worker	33.2 (242)	38.5 (557)
	Non-manual worker	60.0 (437)	52.3 (757)
	Employer-self-employed	5.5 (40)	8.1 (118)
	Missing	1.2 (9)	1.1 (16)
Married/cohabiting	No	33.7 (245)	33.4 (483)
	Yes	66.3 (483)	66.6 (965)
	Missing	---	---
Age at menarche	≤12	20.9 (152)	21.4 (310)
	>12 to <15	52.2 (380)	53.8 (779)
	≥15	26.0 (189)	23.8 (344)
	Missing	1.0 (7)	1.0 (15)
Parity	Nullipara	11.5 (84)	9.7 (141)
	1	19.8 (144)	21.3 (309)
	2	44.6 (325)	41.9 (607)
	≥3	21.6 (157)	24.8 (359)
	Missing	2.5 (18)	2.2 (32)
Age at first childbirth	Nullipara	11.5 (84)	9.7 (141)
	≤20	15.5 (113)	16.2 (234)
	>20 to ≤25	34.8 (253)	36.3 (526)
	>25 to ≤30	25.5 (186)	26.7 (387)
	>30	10.2 (74)	8.8 (127)
	Missing	2.5 (18)	2.3 (33)
Bilateral oophorectomy	No	98.9 (720)	98.3 (1424)
	Yes	1.1 (8)	1.7 (24)
	Missing	---	---
	Pre-/Perimenopausal	33.3 (482)	34.6 (252)
Age at menopause	≤45	11.9 (172)	11.5 (84)
	>45 to <53	39.2 (567)	37.4 (272)
	≥53	14.4 (209)	14.3 (104)
	Missing	1.2 (18)	2.2 (16)
Exposure to OC (ever/never)	No	46.8 (341)	49.0 (709)
	Yes	53.0 (386)	50.8 (735)
	Missing	0.1 (1)	0.3 (4)
Exposure to HRT (current/non)	No, pre menopausal	23.0 (167)	21.1 (306)
	No, peri-/post menopausal	49.3 (359)	59.4 (860)
	ERT	5.1 (37)	6.3 (91)
	PRT	1.2 (9)	0.5 (7)
	CHRT	21.2 (154)	12.3 (178)
	Missing	0.3 (2)	0.4 (6)
Height	*Mean (standard deviation)*	*164.3 (5.8)*	*163.8 (6.0)*
	Missing for both cases and controls		
Body mass index	*Mean (standard deviation)*	*25.6 (4.1)*	*25.5 (4.4)*
	Missing for both cases and controls		
Alcohol consumption	Nothing last year (teetotaler)	10.2 (74)	12.0 (174)
	Something last year (not last month)	10.6 (77)	12.1 (175)
	Something last month	79.0 (575)	75.6 (1094)
	Missing	0.3 (2)	0.3 (5)
Smoking	Never	43.4 (316)	43.2 (626)
	Current	27.5 (200)	28.1 (407)
	Ex	29.1 (212)	28.5 (413)
	Missing	0.0 (0)	0.1 (2)

### Selected SNPs in relation to breast cancer risk

A breast cancer association was seen for six of the 14 tested SNPs; rs2981582 (FGFR2) 1.28 (1.12-1.47), rs3803662 (TNRC9) 1.20 (1.04-1.39), rs12443621 (TNRC9) 1.19 (1.04-1.35), rs889312 (MAP3K1) 1.18 (1.02-1.36), rs3817198 (LSP1) 1.17 (1.10-1.35) and rs2107425 (H19) 0.86 (0.75-0.99) (Table 2).

**Table 2:** Overall breast cancer risk in relation to selected SNPs

**SNP Rs number (Gene)**	**Case/control N**	**Breast cancer risk Crude OR (CI 95%)**	Breast cancer risk Adjusted OR* (CI 95%)	Breast cancer risk Adjusted OR** (CI 95%)
**rs2981582 (FGFR2)**				
CC	233/561	1.00	1.00	1.00
CT	356/653	1.31 (1.08-1.60)	1.31 (1.07-1.60)	1.31 (1.07-1.61)
TT	124/185	1.61 (1.23-2.12)	1.62 (1.23-2.13)	1.63 (1.23-2.16)
**Per allele**		**1.28 (1.12-1.46)**	**1.28 (1.12-1.46)**	**1.28 (1.12-1.47)**
**rs1045485 (CASP8)**				
CC	185/374	1.00	1.00	1.00
CG	42/86	0.99 (0.66-1.49)	0.98 (0.65-1.48)	1.04 (0.69-1.58)
GG	8/10	1.62 (0.63-4.17)	1.61 (0.63-4.16)	1.73 (0.66-4.54)
**Per allele**		**1.10 (0.79-1.51)**	**1.10 (0.80-1.54)**	**1.10 (0.79-1.54)**
**rs3803662 (TNRC9)**				
CC	353/780	1.00	1.00	1.00
CT	278/512	1.20 (0.99-1.46)	1.20 (0.99-1.46)	1.19 (0.98-1.45)
TT	64/95	1.49 (1.06-2.09)	1.49 (1.06-2.10)	1.47 (1.04-2.08)
**Per allele**		**1.21 (1.05-1.40)**	**1.21 (1.05-1.40)**	**1.20 (1.04-1.39)**
**rs8051542 (TNRC9)**				
CC	192/443	1.00	1.00	1.00
CT	338/637	1.22 (0.99-1.52)	1.23 (0.99-1.52)	1.20 (0.97-1.50)
TT	149/272	1.26 (0.97-1.64)	1.27 (0.97-1.64)	1.24 (0.95-1.62)
**Per allele**		**1.13 (1.00-1.29)**	**1.13 (1.00-1.29)**	**1.12 (0.99-1.28)**
**rs12443621 (TNRC9)**				
AA	195/451	1.00	1.00	1.00
AG	338/657	1.19 (0.96-1.47)	1.19 (0.96-1.47)	1.20 (0.97-1.49)
GG	165/275	1.39 (1.07-1.79)	1.39 (1.08-1.79)	1.42 (1.09-1.84)
**Per allele**		**1.18 (1.04-1.34)**	**1.18 (1.04-1.34)**	**1.19 (1.04-1.35)**
**rs889312 (MAP3K1)**				
AA	322/737	1.00	1.00	1.00
AC	301/530	1.30 (1.07-1.58)	1.30 (1.07-1.58)	1.26 (1.03-1.53)
CC	66/118	1.28 (0.92-1.78)	1.28 (0.92-1.78)	1.29 (0.92-1.80)
**Per allele**		**1.19 (1.04-1.37)**	**1.19 (1.04-1.37)**	**1.18 (1.02-1.36)**
**rs3817198 (LSP1)**				
TT	311/668	1.00	1.00	1.00
CT	282/555	1.09 (0.90-1.33)	1.09 (0.90-1.33)	1.06 (0.87-1.30)
CC	76/107	1.53 (1.10-2.11)	1.53 (1.11-2.11)	1.50 (1.08-2.09)
**Per allele**		**1.18 (1.02-1.36)**	**1.18 (1.02-1.36)**	**1.17 (1.10-1.35)**
**rs2107425 (H19)**				
CC	361/637	1.00	1.00	1.00
CT	250/573	0.77 (0.63-0.94)	0.77 (0.63-0.94)	0.78 (0.64-0.95)
TT	68/145	0.83 (0.60-1.14)	0.83 (0.60-1.14)	0.83 (0.60-1.14)
**Per allele**		**0.86 (0.74-0.99)**	**0.86 (0.74-0.99)**	**0.86 (0.75-0.99)**
**rs13281615 (8q24)**				
AA	245/533	1.00	1.00	1.00
AG	332/633	1.14 (0.93-1.40)	1.14 (0.93-1.40)	1.14 (0.93-1.40)
GG	117/204	1.25 (0.95-1.64)	1.25 (0.95-1.64)	1.25 (0.95-1.64)
**Per allele**		**1.12 (0.98-1.28)**	**1.12 (0.98-1.28)**	**1.15 (1.00-1.31)**
**rs981782 (5p12)**				
TT	182/335	1.00	1.00	1.00
TG	352/685	0.95 (0.76-1.18)	0.95 (0.76-1.18)	0.91 (0.72-1.14)
GG	125/296	0.78 (0.59-1.03)	0.78 (0.59-1.03)	0.74 (0.56-0.98)
**Per allele**		**0.89 (0.77-1.02)**	**0.89 (0.77-1.02)**	**0.87 (0.75-1.00)**
**rs30099 (5q)**				
CC	584/1139	1.00	1.00	1.00
CT	113/248	0.89 (0.70-1.13)	0.89 (0.70-1.14)	0.90 (0.71-1.16)
TT	11/12	1.79 (0.78-4.08)	1.79 (0.78-4.10)	1.79 (0.77-4.18)
**Per allele**		**0.98 (0.79-1.21)**	**0.98 (0.79-1.22)**	**0.99 (0.80-1.24)**
**rs4666451 (2p)**				
GG	272/554	1.00	1.00	1.00
GA	299/574	1.06 (0.87-1.30)	1.06 (0.87-1.30)	1.08 (0.88-1.32)
AA	105/204	1.05 (0.80-1.38)	1.05 (0.79-1.38)	1.06 (0.80-1.40)
**Per allele**		**1.03 (0.91-1.18)**	**1.03 (0.91-1.18)**	**1.04 (0.91-1.19)**
**rs13387042 (2q35)**				
AA	192/335	1.00	1.00	1.00
AG	330/657	1.08 (0.86-1.36)	1.08 (0.86-1.35)	1.08 (0.86-1.37)
GG	163/350	1.23 (0.95-1.59)	1.23 (0.95-1.59)	1.24 (0.96-1.62)
**Per allele**		**0.90 (0.79-1.03)**	**0.90 (0.79-1.03)**	**0.90 (0.79-1.02)**
**rs7766585 (ESR1)**				
CC	518/1031	1.00	1.00	1.00
CT	172/348	0.98 (0.80-1.22)	0.98 (0.80-1.22)	0.98 (0.79-1.21)
TT	17/26	1.30 (0.70-2.42)	1.30 (0.70-2.42)	1.42 (0.76-2.67)
**Per allele**		**1.03 (0.86-1.23)**	**1.03 (0.86-1.23)**	**1.03 (0.86-1.24)**

* Adjusted for matching variables (age and year of inclusion in study).

** Adjusted for matching variables (age and year of inclusion in study), and for selected confounders (socioeconomic status and exposure to HRT).

### Selected reproductive risk factors in relation to breast cancer risk

In the analyses of breast cancer risk associated with the studied reproductive factors, we could see a borderline decreased breast cancer risk for parous women as compared to nulliparous women RR: 0.82 (0.62-1.10). For age at first birth, there was no breast cancer association (>25 years of age RR: 1.05 (0.87-1.28)).

### Selected SNPs and breast cancer risk according to parity and age at first birth

Only one interaction between the effects of the tested SNPs (rs981782 (5p12)) and parity was found with regard to breast cancer risk p = 0.02 (Table 3). This association was not seen when stratifying on four parity groups (Additional file 1: Appendix1). No association between the tested SNPs and age at first birth was seen with regard to breast cancer risk. Moreover, no statistically significant interaction between the effects of tested SNPs and parity/age at first childbirth on breast cancer risk was seen after adjusting for multiple comparisons (using the corrected p-value cut-off <0.0018).

**Table 3:** Breast cancer risk in relation to selected SNPs with regard to parity

**Rs nr (Gene)**	**p-value inter-action**	**Case/ control N**	Nulliparous OR* (CI 95%)	**Case/ control N**	Parous OR* (CI 95%)
**rs2981582 (FGFR2)**	0.96				
CC		30/52	1.00	200/493	1.00
CT		36/69	0.90 (0.48-1.69)	310/571	1.36 (1.09-1.69)
TT		16/15	1.88 (0.78-4.52)	104/167	1.55 (1.15-2.10)
**Per allele**			**1.25 (0.82-1.91)**		**1.27 (1.10-1.46)**
**rs1045485 (CASP8)**	0.93				
CC		21/48	1.00	162/318	1.00
CG		7/14	1.08 (0.37-3.18)	35/71	1.03 (0.65-1.63)
GG		0/0	---	8/10	1.68 (0.64-4.42)
**Per allele**			**0.93 (0.31-2.83)**		**1.12 (0.79-1.58)**
**rs3803662 (TNRC9)**	0.58				
CC		36/72	1.00	311/694	1.00
CT		33/54	1.32 (0.71-2.44)	238/446	1.17 (0.95-1.47)
TT		8/7	1.95 (0.61-6.29)	54/82	1.47 (1.01-2.14)
**Per allele**			**1.33 (0.83-2.14)**		**1.19 (1.02-1.40)**
**rs8051542 (TNRC9)**	0.15				
CC		14/38	1.00	177/399	1.00
CT		41/66	1.56 (0.73-3.36)	289/558	1.14 (0.91-1.44)
TT		23/28	2.18 (0.93-5.14)	120/234	1.14 (0.86-1.52)
**Per allele**			**1.47 (0.96-2.24)**		**1.08 (0.94-1.24)**
**rs12443621 (TNRC9)**	0.95				
AA		22/30	1.00	170/415	1.00
AG		35/79	0.68 (0.33-1.42)	295/565	1.28 (1.02-1.62)
GG		25/25	1.55 (0.67-3.61)	135/237	1.43 (1.08-1.89)
**Per allele**			**1.25 (0.82-1.92)**		**1.20 (1.04-1.38)**
**rs889312 (MAP3K1)**	0.11				
AA		42/67	1.00	273/653	1.00
AC		33/59	0.83 (0.46-1.53)	261/461	1.31 (1.06-1.62)
CC		4/10	0.49 (0.14-1.77)	61/103	1.47 (1.03-2.09)
**Per allele**			**0.77 (0.48-1.24)**		**1.24 (1.07-1.45)**
**rs3817198 (LSP1)**	0.48				
TT		37/68	1.00	271/591	1.00
CT		31/55	1.12 (0.60-2.11)	239/482	1.06 (0.85-1.31)
CC		8/4	4.38 (1.13-16.96)	68/100	1.48 (1.04-207)
**Per allele**			**1.53 (0.93-2.51)**		**1.16 (0.99-1.35)**
**rs2107425 (H19)**	0.12				
CC		44/57	1.00	311/570	1.00
CT		27/57	0.54 (0.28-1.05)	213/498	0.80 (0.65-1.00)
TT		4/14	0.35 (0.11-1.19)	64/127	0.94 (0.67-1.31)
**Per allele**			**0.58 (0.36-0.95)**		**0.91 (0.78-1.06)**
**rs13281615 (8q24)**	1.00				
AA		29/52	1.00	211/465	1.00
AG		37/61	1.11 (0.58-2.15)	290/558	1.18 (0.95-1.47)
GG		13/19	1.32 (0.55-3.15)	99/183	1.26 (0.93-1.70)
**Per allele**			**1.14 (0.75-1.73)**		**1.13 (0.98-1.31)**
**rs981782 (5p12)**	0.02				
TT		15/38	1.00	162/290	1.00
TG		43/72	1.67 (0.79-3.53)	303/595	0.85 (0.67-1.09)
GG		14/16	2.66 (0.99-7.12)	107/273	0.65 (0.48-0.87)
**Per allele**			**1.64 (1.01-2.67)**		**0.81 (0.70-0.94)**
**rs30099 (5q)**	0.47				
CC		66/101	1.00	505/1015	1.00
CT		14/34	0.68 (0.32-1.43)	97/205	0.95 (0.73-1.24)
TT		2/1	3.18 (0.26-39.46)	9/11	1.63 (0.66-4.06)
**Per allele**			**0.89 (0.47-1.67)**		**1.02 (0.81-1.30)**
**rs4666451 (2p)**	0.37				
GG		26/51	1.00	237/494	1.00
GA		38/54	1.39 (0.69-2.59)	256/502	1.08 (0.87-1.34)
AA		15/20	1.30 (0.54-3.10)	88/180	1.02 (0.75-1.38)
**Per allele**			**1.17 (0.77-1.79)**		**1.02 (0.89-1.18)**
**rs13387042 (2q35)**	0.79				
AA		22/35	1.00	164/292	1.00
AG		40/60	1.11 (0.55-2.25)	287/580	0.87 (0.68-1.11)
GG		16/36	0.69 (0.30-1.61)	140/308	0.80 (0.60-1.06)
**Per allele**			**0.85 (0.56-1.28)**		**0.89 (0.78-1.03)**
**rs7766585 (ESR1)**	0.10				
CC		63/97	1.00	443/907	1.00
CT		18/33	0.76 (0.39-1.51)	150/310	0.99 (0.79-1.24)
TT		0/6	----	17/20	1.91 (0.98-3.71)
**Per allele**			**0.96 (0.82-1.12)**		**1.03 (0.97-1.10)**

*Adjusted for: age, year of inclusion in study, socioeconomic status and exposure to HRT.

In the per allele analyses, no clear patterns for risk associations were seen in the stratified analyses (Table 3, Table 4, Additional file 1: Appendix 1 and Appendix 2).

**Table 4:** Breast cancer risk in relation to selected SNPs with regard to age at first child-birth

**Rs nr (Gene)**	**p-value inter-action**	**Case/ control N**	Age < 25 years OR* (CI 95%)	**Case/ control N**	Age > 25 years OR* (CI 95%)
**rs2981582 (FGFR2)**	0.47				
CC		110/291	1.00	90/202	1.00
CT		191/352	1.49 (1.20-1.99)	119/219	1.18 (0.84-1.66)
TT		60/95	1.68 (1.13-2.51)	44/71	1.38 (0.87-2.20)
**Per allele**			**1.33 (1.10-1.61)**		**1.17 (0.94-1.47)**
**rs1045485 (CASP8)**	0.26				
CC		104/192	1.00	58/126	1.00
CG		21/41	0.99 (0.55-1.80)	14/30	1.14 (0.55-2.34)
GG		3/7	0.88 (0.22-3.60)	5/3	3.21 (0.71-14.61)
**Per allele**			**0.93 (0.58-1.49)**		**1.38 (0.79-2.42)**
**rs3803662 (TNRC9)**	0.91				
CC		185/419	1.00	126/275	1.00
CT		143/257	1.25 (0.95-1.37)	95/188	1.08 (0.78-1.51)
TT		31/53	1.32 (0.81-2.16)	23/29	1.72 (0.94-3.12)
**Per allele**			**1.19 (0.97-1.46)**		**1.21 (0.94-1.55)**
**rs8051542 (TNRC9)**	0.48				
CC		112/256	1.00	65/143	1.00
CT		166/313	1.23 (0.91-1.65)	123/244	1.06 (0.73-1.53)
TT		68/149	1.01 (0.70-1.46)	52/85	1.36 (0.85-2.16)
**Per allele**			**1.03 (0.86-1.23)**		**1.16 (0.92-1.46)**
**rs12443621 (TNRC9)**	0.82				
AA		104/269	1.00	66/146	1.00
AG		169/312	1.48 (1.10-2.01)	126/252	1.07 (0.74-1.55)
GG		79/146	1.45 (1.01-2.08)	56/91	1.39 (0.88-2.18)
**Per allele**			**1.22 (1.02-1.46)**		**1.17 (0.93-1.46)**
**rs889312 (MAP3K1)**	0.65				
AA		153/371	1.00	120/282	1.00
AC		161/288	1.30 (0.99-1.71)	100/172	1.33 (0.95-1.86)
CC		37/68	1.38 (0.88-2.16)	24/35	1.61 (0.91-2.86)
**Per allele**			**1.21 (1.00-1.48)**		**1.30 (1.02-1.65)**
**rs3817198 (LSP1)**	0.17				
TT		160/359	1.00	111/232	1.00
CT		139/287	1.05 (0.79-1.39)	100/195	1.04 (0.74-1.46)
CC		44/50	2.00 (1.27-3.14)	24/49	1.00 (0.58-1.73)
**Per allele**			**1.27 (1.04-1.55)**		**1.02 (0.80-1.30)**
**rs2107425 (H19)**	0.09				
CC		191/329	1.00	120/240	1.00
CT		120/304	0.69 (0.52-0.91)	93/194	0.99 (0.71-1.39)
TT		35/78	0.78 (0.50-1.22)	29/49	1.19 (0.71-2.00)
**Per allele**			**0.81 (0.66-0.99)**		**1.06 (0.83-1.33)**
**rs13281615 (8q24)**	0.80				
AA		124/290	1.00	87/175	1.00
AG		172/323	1.27 (0.95-1.69)	118/234	1.04 (0.74-1.48)
GG		54/107	1.25 (0.84-1.86)	45/76	1.27 (0.80-2.01)
**Per allele**			**1.15 (0.95-1.83)**		**1.11 (0.89-1.39)**
**rs981782 (5p12)**	0.38				
TT		88/173	1.00	74/117	1.00
TG		185/351	0.96 (0.70-1.32)	118/243	0.73 (0.50-1.06)
GG		67/169	0.71 (0.48-1.05)	40/104	0.60 (0.37-0.96)
**Per allele**			**0.85 (0.70-1.03)**		**0.77 (0.61-0.98**
**rs30099 (5q)**	0.31				
CC		299/621	1.00	206/393	1.00
CT		54/108	1.02 (0.71-1.46)	43/97	0.83 (0.55-1.25)
TT		6/6	2.47 (0.77-7.91)	3/5	0.76 (0.16-3.62)
**Per allele**			**1.14 (0.83-1.56)**		**0.85 (0.59-1.23)**
**rs4666451 (2p)**	0.24				
GG		131/301	1.00	106/192	1.00
GA		159/295	1.23 (0.92-1.64)	97/207	0.89 (0.63-1.26)
AA		53/109	1.11 (0.75-1.65)	35/71	0.90 (0.56-1.45)
**Per allele**			**1.09 (0.91-1.31)**		**0.93 (0.74-1.17)**
**rs13387042 (2q35)**	0.89				
AA		95/168	1.00	69/124	1.00
AG		170/348	0.86 (0.63-1.19)	117/232	0.89 (0.61-1.30)
GG		84/186	0.80 (0.55-1.15)	56/121	0.84 (0.54-1.31)
**Per allele**			**0.89 (0.74-1.07)**		**0.92 (0.74-1.14)**
**rs7766585 (ESR1)**	0.60				
CC		249/525	1.00	194/382	1.00
CT		99/199	1.07 (0.80-1.43)	51/110	0.88 (0.60-1.29)
TT		11/13	2.06 (0.89-4.75)	6/7	1.68 (0.55-5.19)
**Per allele**			**1.07 (0.98-1.18)**		**0.98 (0.90-1.08)**

*Adjusted for: age, year of inclusion in study, socioeconomic status and exposure to HRT.

### Sensitivity analysis including subjects with information on at least 80% of SNPs

When including only women with information on at least 80% of SNPs, the results were fairly similar in all analyses but some analyses with borderline significant ORs became significant when only individuals with information on ≥ 80% of all SNPs were analysed (data not shown).

## Discussion

The results of this present study are in agreement with previous GWAS studies for six out of 14 SNPs. With respect to breast cancer risk, there were no statistically significant gene-environment interactions between parity/age at first childbirth and SNPs and this is in agreement with the results in three other large-scale investigations [12-14].

### Methodological considerations

#### Representativity

A total of 40% of the invited women in Malmö participated in the MDCS and women in the MDCS have been shown to have a higher incidence of breast cancer and they may also be selected towards a slightly higher socioeconomic status [8] than the general population. However, as the present study use internal comparisons, yielding relative risks rather than incidence rates, the impact of a potential selection bias was probably limited.

### Reproductive factors

Parity and age at first birth were the main exposures of this study and were obtained from questionnaires answered at baseline. All women were 44 years or older at baseline, hence unlikely to have given birth to more children thereafter.

### SNP analyses

SNP analysis method has been validated by repeating the analyses twice, in a subset and the reproducibility was very high [11]. In order to verify that the results were not altered by damaged DNA, the analyses were repeated including only women with results on 80% or more of the SNP analyses. Following this, all results were similar.

### Statistical power

Overall, the sample size of this study was fairly small, yielding a statistical power issue. Many comparisons were made and there is a potential risk of a type I error. The replication of results concerning selected SNPs and breast cancer risk was based on previous studies, and all but one SNP showed associations in the expected direction (statistically significant for six out of 14 SNPs). This strengthens the assumption that these results reflect true associations and were not only the result of multiple comparisons. As these analyses are made with an a priori hypothesis, the Bonferroni correction for multiple testing was not considered relevant. Concerning interaction analyses and the stratified analyses, these analyses were exploratory and hypothesis generating hence corrections for multiple comparisons for the performed interactions was considered valid. Due to few individuals in the analyses, the confidence intervals were wide and the statistical power was low which can have lead to a type II error. In order to address the risk of type II error, parity and age at first childbirth were dichotomized yielding larger study groups. Moreover, interaction term corrected for multiple comparisons (Bonferroni correction) yielded no statistically significant interactions.

### Previous studies

To our knowledge, four studies have been published studying breast cancer risk and the potential interaction between SNPs and parity/age at first childbirth [12-15]. The SNP rs2981582 (FGR2), was studied by Kawase et al. and they found a high breast cancer risk for nulliparous women and for women giving birth to one or two children, carrying homozygote minor allele of rs2981582 (FGR2). In their study, a total of 456 cases and 912 controls were included which is comparable to the present study; however they only included one SNP [15].

The study by Travis et al. examined 120 gene-environmental interactions (i.e. reproductive, behavioural, and anthropometric risk factors for breast cancer) categorising parity as nulliparous vs. parous and age at first childbirth as younger or older than 25 years of age in 7610 cases and 10 196 controls, making the results less vulnerable to type II error. They studied 12 SNPs and did not find any statistically significant interaction. Four of the SNPs examined in the present study (rs8051542 (TNRC9), rs12443621 (TNRC9), rs2107425 (H19) and rs7766585 (ESR1)) were not studied by Travis et al. [12].

Milne et al. studied ten GWAS SNPs and two candidate SNPs associated with breast cancer in 26349 invasive breast cancer cases and 32208 controls with regard to interaction with reproductive factors. After adjustment for multiple comparisons no significant association was seen for parity (continuous and categorical) or age at first childbirth (continuous and categorical). Five of the studied SNPs by Milne et al. are the same as in this study: rs2981582 (FGFR2), rs889312 (MAP3K1), rs3817198 (LSP1), rs13281615 (8q24) and rs13387042 (2q35). The other seven SNPs studied by Milne et al. have been studied in more recent GWAS studies and are hence not included in the present study [13]. Due to differences in SNP selection, all results from this study may not be comparable to the results of Milne et al.. However, three of the SNPs studied by Milne et al. are from same genes as in this study 5p12, CASP8 and ESR1.

Campa et al. studied 17 SNPs associated with breast cancer risk. When analysing gene-environmental risks including parity and other reproductive factors, no statistically significant association was seen. The study of Campa et al. included 8575 cases and 11892 controls, making this study less vulnerable to type II errors. Seven of the studied SNPs of Campa et al are the same as in the present study and one is on the same gene, making some of the results comparable to the present study.

## Conclusions

The results of this present study are in agreement with previous GWAS studies in SNPs and breast cancer risk for six out of 14 risk SNPs and is in agreement of null results for SNP parity/age at first childbirth interaction.

## Competing interests

None of the authors have declared any competing interests.

## Authors’ contribution

SB carried out all the statistical analyses, participated in interpreting the results, reviewed the literature and drafted the manuscript. SH performed the SNP analyses, participated in interpreting the results and critically revised the manuscript. SiB participated in interpreting the results and critically revised the manuscript. MI performed the SNP analyses and critically revised the manuscript. GL participated in interpreting the results and critically revised the manuscript. JD participated in designing the study, critically revised the statistical methods and the manuscript. JC participated in designing the study and critically revised the results and the manuscript. JM designed the study, supervised and participated in interpreting all statistical analyses and critically revised the manuscript. All authors have read and given approval of the final manuscript.

## Availability of supporting data

The study was carried out in the MDCS. In order to get access to the data, an application to the committee is needed. Please contact Ass. Prof. Jonas Manjer: jonas.manjer@med.lu.se.

## Supplementary Material

Additional file 1 **Appendix 1.** Breast cancer risk in relation to selected SNPs stratified on parity. **Appendix 2.** Breast cancer risk in relation to selected SNPs stratified on age at first child-birth. Click here for file

## References

[B1] Familial breast cancer: collaborative reanalysis of individual data from 52 epidemiological studies including 58,209 women with breast cancer and 101,986 women without the disease Lancet 2001 358 9291 1389 1399 11705483 10.1016/S0140-6736(01)06524-2

[B2] Lux MP Fasching PA Beckmann MW Hereditary breast and ovarian cancer: review and future perspectives J Mol Med 2006 84 1 16 28 10.1007/s00109-005-0696-7 16283147

[B3] Parsa P Parsa B Effects of reproductive factors on risk of breast cancer: a literature review Asian Pac J Cancer Prev 2009 10 4 545 550 19827866

[B4] Easton DF Pooley KA Dunning AM Pharoah PD Thompson D Ballinger DG Genome-wide association study identifies novel breast cancer susceptibility loci Nature 2007 447 7148 1087 1093 10.1038/nature05887 17529967 PMC2714974

[B5] Stacey SN Manolescu A Sulem P Rafnar T Gudmundsson J Gudjonsson SA Common variants on chromosomes 2q35 and 16q12 confer susceptibility to estrogen receptor-positive breast cancer Nat Genet 2007 39 7 865 869 10.1038/ng2064 17529974

[B6] Stacey SN Manolescu A Sulem P Thorlacius S Gudjonsson SA Jonsson GF Common variants on chromosome 5p12 confer susceptibility to estrogen receptor-positive breast cancer Nat Genet 2008 40 6 703 706 10.1038/ng.131 18438407

[B7] Cox A Dunning AM Garcia-Closas M Balasubramanian S Reed MW Pooley KA A common coding variant in CASP8 is associated with breast cancer risk Nat Genet 2007 39 3 352 358 10.1038/ng1981 17293864

[B8] Manjer J Carlsson S Elmstahl S Gullberg B Janzon L Lindstrom M The Malmo Diet and Cancer Study: representativity, cancer incidence and mortality in participants and non-participants Eur J Cancer Prev 2001 10 6 489 499 10.1097/00008469-200112000-00003 11916347

[B9] Manjer J Elmstahl S Janzon L Berglund G Invitation to a population-based cohort study: differences between subjects recruited using various strategies Scand J Public Health 2002 30 2 103 112 12028859 10.1177/14034948020300020401

[B10] Butt S Borgquist S Anagnostaki L Landberg G Manjer J Parity and age at first childbirth in relation to the risk of different breast cancer subgroups Int J Cancer 2009 125 8 1926 1934 10.1002/ijc.24494 19569233

[B11] Harlid S Ivarsson MI Butt S Shehnaz H Grybowska E Eyfjörð JE A Candidate CpG SNP Approach Identifies a Breast Cancer associated ESR1-allele Int J Cancer 2011 129 7 1689 1698 10.1002/ijc.25786 21105050

[B12] Travis RC Reeves GK Green J Bull D Tipper SJ Baker K Gene-environment interactions in 7610 women with breast cancer: prospective evidence from the Million Women Study Lancet 2010 375 9732 2143 2151 10.1016/S0140-6736(10)60636-8 20605201 PMC2890858

[B13] Milne RL Gaudet MM Spurdle AB Fasching PA Couch FJ Benitez J Assessing interactions between the associations of common genetic susceptibility variants, reproductive history and body mass index with breast cancer risk in the breast cancer association consortium: a combined case–control study Breast Cancer Res 2010 12 6 R110 10.1186/bcr2797 21194473 PMC3046455

[B14] Campa D Kaaks R Le Marchand L Haiman CA Travis RC Berg CD Interactions between genetic variants and breast cancer risk factors in the breast and prostate cancer cohort consortium J Natl Cancer Inst 2011 103 16 1252 1263 10.1093/jnci/djr265 21791674 PMC3156803

[B15] Kawase T Matsuo K Suzuki T Hiraki A Watanabe M Iwata H FGFR2 intronic polymorphisms interact with reproductive risk factors of breast cancer: results of a case control study in Japan Int J Cancer 2009 125 8 1946 1952 10.1002/ijc.24505 19582883

