# Supplementary material for: Genetic predisposition, parity, age at first childbirth and risk for breast cancer
Source: BMC Res Notes. 2012 Aug 7;5:414. doi: 10.1186/1756-0500-5-414 (PMC3439270; doi:10.1186/1756-0500-5-414)
Supplement: Additional file 1 — Appendix 1. Breast cancer risk in relation to selected SNPs stratified on parity. Appendix 2. Breast cancer risk in relation to selected SNPs stratified on age at first child-birth. [file 1756-0500-5-414-S1.doc]

Table 1 Case-Control status and distribution of potential confounders

| **Factor** | **Category** | **Case**  **(n=728)** | **Control**  **(n=1448)** |
| --- | --- | --- | --- |
|  |  | Column % (n)  *Mean (SD) in italics* | |
| Education | O-level college | 67.9 (494) | 70.2 (1017) |
|  | A-level college | 7.1 (52) | 7.0 (101) |
|  | University | 24.7 (180) | 22.4 (325) |
|  | Missing | 0.3 (2) | 0.3 (5) |
|  |  |  |  |
| Type of occupation | Manual worker | 33.2 (242) | 38.5 (557) |
|  | Non-manual worker | 60.0 (437) | 52.3 (757) |
|  | Employer-self-employed | 5.5 (40) | 8.1 (118) |
|  | Missing | 1.2 (9) | 1.1 (16) |
|  |  |  |  |
| Married/cohabiting | No | 33.7 (245) | 33.4 (483) |
|  | Yes | 66.3 (483) | 66.6 (965) |
|  | Missing | --- | --- |
|  |  |  |  |
| Age at menarche | <12 | 20.9 (152) | 21.4 (310) |
|  | >12 to <15 | 52.2 (380) | 53.8 (779) |
|  | >15 | 26.0 (189) | 23.8 (344) |
|  | Missing | 1.0 (7) | 1.0 (15) |
|  |  |  |  |
| Parity | Nullipara | 11.5 (84) | 9.7 (141) |
|  | 1 | 19.8 (144) | 21.3 (309) |
|  | 2 | 44.6 (325) | 41.9 (607) |
|  | >3 | 21.6 (157) | 24.8 (359) |
|  | Missing | 2.5 (18) | 2.2 (32) |
|  |  |  |  |
| Age at first childbirth | Nullipara | 11.5 (84) | 9.7 (141) |
|  | <20 | 15.5 (113) | 16.2 (234) |
|  | >20 to <25 | 34.8 (253) | 36.3 (526) |
|  | >25 to <30 | 25.5 (186) | 26.7 (387) |
|  | >30 | 10.2 (74) | 8.8 (127) |
|  | Missing | 2.5 (18) | 2.3 (33) |
|  |  |  |  |
| Bilateral oophorectomy | No | 98.9 (720) | 98.3 (1424) |
|  | Yes | 1.1 (8) | 1.7 (24) |
|  | Missing | --- | --- |
|  |  |  |  |
|  | Pre-/Perimenopausal | 33.3 (482) | 34.6 (252) |
| Age at menopause | <45 | 11.9 (172) | 11.5 (84) |
|  | >45 to <53 | 39.2 (567) | 37.4 (272) |
|  | >53 | 14.4 (209) | 14.3 (104) |
|  | Missing | 1.2 (18) | 2.2 (16) |
|  |  |  |  |
| Exposure to OC | No | 46.8 (341) | 49.0 (709) |
| (ever/never) | Yes | 53.0 (386) | 50.8 (735) |
|  | Missing | 0.1 (1) | 0.3 (4) |
|  |  |  |  |
| Exposure to HRT | No, pre menopausal | 23.0 (167) | 21.1 (306) |
| (current/non) | No, peri-/post menopausal | 49.3 (359) | 59.4 (860) |
|  | ERT | 5.1 (37) | 6.3 (91) |
|  | PRT | 1.2 (9) | 0.5 (7) |
|  | CHRT | 21.2 (154) | 12.3 (178) |
|  | Missing | 0.3 (2) | 0.4 (6) |
|  |  |  |  |
| Height | *Mean (standard deviation)* | *164.3 (5.8)* | *163.8 (6.0)* |
|  | Missing |  |  |
|  |  |  |  |
| Body mass index | *Mean (standard deviation)* | *25.6 (4.1)* | *25.5 (4.4)* |
|  | Missing |  |  |
|  |  |  |  |
| Alcohol consumption | Nothing last year (teetotaler) | 10.2 (74) | 12.0 (174) |
|  | Something last year (not last month) | 10.6 (77) | 12.1 (175) |
|  | Something last month | 79.0 (575) | 75.6 (1094) |
|  | Missing | 0.3 (2) | 0.3 (5) |
|  |  |  |  |
| Smoking | Never | 43.4 (316) | 43.2 (626) |
|  | Current | 27.5 (200) | 28.1 (407) |
|  | Ex | 29.1 (212) | 28.5 (413) |
|  | Missing | 0.0 (0) | 0.1 (2) |

**Table 2 Overall breast cancer risk in relation to selected SNPs. Odds ratios (OR) with 95% confidence intervals (CI)**

| **SNP**  **Rs number (Gene)** | **Case/control**  **N** | **Breast cancer risk**  **Crude OR (CI 95%)** | **Breast cancer risk**  **Adjusted OR* (CI 95%)** | **Breast cancer risk**  **Adjusted OR** (CI 95%)** |
| --- | --- | --- | --- | --- |
| **rs2981582 (FGFR2)** |  |  |  |  |
| CC | 233/561 | 1.00 | 1.00 | 1.00 |
| CT | 356/653 | 1.31 (1.08-1.60) | 1.31 (1.07-1.60) | 1.31 (1.07-1.61) |
| TT | 124/185 | 1.61 (1.23-2.12) | 1.62 (1.23-2.13) | 1.63 (1.23-2.16) |
| **Per allele** |  | **1.28 (1.12-1.46)** | **1.28 (1.12-1.46)** | **1.28 (1.12-1.47)** |
| **rs1045485 (CASP8)** |  |  |  |  |
| CC | 185/374 | 1.00 | 1.00 | 1.00 |
| CG | 42/86 | 0.99 (0.66-1.49) | 0.98 (0.65-1.48) | 1.04 (0.69-1.58) |
| GG | 8/10 | 1.62 (0.63-4.17) | 1.61 (0.63-4.16) | 1.73 (0.66-4.54) |
| **Per allele** |  | **1.10 (0.79-1.51)** | **1.10 (0.80-1.54)** | **1.10 (0.79-1.54)** |
| **rs3803662 (TNRC9)** |  |  |  |  |
| CC | 353/780 | 1.00 | 1.00 | 1.00 |
| CT | 278/512 | 1.20 (0.99-1.46) | 1.20 (0.99-1.46) | 1.19 (0.98-1.45) |
| TT | 64/95 | 1.49 (1.06-2.09) | 1.49 (1.06-2.10) | 1.47 (1.04-2.08) |
| **Per allele** |  | **1.21 (1.05-1.40)** | **1.21 (1.05-1.40)** | **1.20 (1.04-1.39)** |
| **rs8051542 (TNRC9)** |  |  |  |  |
| CC | 192/443 | 1.00 | 1.00 | 1.00 |
| CT | 338/637 | 1.22 (0.99-1.52) | 1.23 (0.99-1.52) | 1.20 (0.97-1.50) |
| TT | 149/272 | 1.26 (0.97-1.64) | 1.27 (0.97-1.64) | 1.24 (0.95-1.62) |
| **Per allele** |  | **1.13 (1.00-1.29)** | **1.13 (1.00-1.29)** | **1.12 (0.99-1.28)** |
| **rs12443621 (TNRC9)** |  |  |  |  |
| AA | 195/451 | 1.00 | 1.00 | 1.00 |
| AG | 338/657 | 1.19 (0.96-1.47) | 1.19 (0.96-1.47) | 1.20 (0.97-1.49) |
| GG | 165/275 | 1.39 (1.07-1.79) | 1.39 (1.08-1.79) | 1.42 (1.09-1.84) |
| **Per allele** |  | **1.18 (1.04-1.34)** | **1.18 (1.04-1.34)** | **1.19 (1.04-1.35)** |
| **rs889312 (MAP3K1)** |  |  |  |  |
| AA | 322/737 | 1.00 | 1.00 | 1.00 |
| AC | 301/530 | 1.30 (1.07-1.58) | 1.30 (1.07-1.58) | 1.26 (1.03-1.53) |
| CC | 66/118 | 1.28 (0.92-1.78) | 1.28 (0.92-1.78) | 1.29 (0.92-1.80) |
| **Per allele** |  | **1.19 (1.04-1.37)** | **1.19 (1.04-1.37)** | **1.18 (1.02-1.36)** |
| **rs3817198 (LSP1)** |  |  |  |  |
| TT | 311/668 | 1.00 | 1.00 | 1.00 |
| CT | 282/555 | 1.09 (0.90-1.33) | 1.09 (0.90-1.33) | 1.06 (0.87-1.30) |
| CC | 76/107 | 1.53 (1.10-2.11) | 1.53 (1.11-2.11) | 1.50 (1.08-2.09) |
| **Per allele** |  | **1.18 (1.02-1.36)** | **1.18 (1.02-1.36)** | **1.17 (1.10-1.35)** |
| **rs2107425 (H19)** |  |  |  |  |
| CC | 361/637 | 1.00 | 1.00 | 1.00 |
| CT | 250/573 | 0.77 (0.63-0.94) | 0.77 (0.63-0.94) | 0.78 (0.64-0.95) |
| TT | 68/145 | 0.83 (0.60-1.14) | 0.83 (0.60-1.14) | 0.83 (0.60-1.14) |
| **Per allele** |  | **0.86 (0.74-0.99)** | **0.86 (0.74-0.99)** | **0.86 (0.75-0.99)** |
| **rs13281615 (8q24)** |  |  |  |  |
| AA | 245/533 | 1.00 | 1.00 | 1.00 |
| AG | 332/633 | 1.14 (0.93-1.40) | 1.14 (0.93-1.40) | 1.14 (0.93-1.40) |
| GG | 117/204 | 1.25 (0.95-1.64) | 1.25 (0.95-1.64) | 1.25 (0.95-1.64) |
| **Per allele** |  | **1.12 (0.98-1.28)** | **1.12 (0.98-1.28)** | **1.15 (1.00-1.31)** |
| **rs981782 (5p12)** |  |  |  |  |
| TT | 182/335 | 1.00 | 1.00 | 1.00 |
| TG | 352/685 | 0.95 (0.76-1.18) | 0.95 (0.76-1.18) | 0.91 (0.72-1.14) |
| GG | 125/296 | 0.78 (0.59-1.03) | 0.78 (0.59-1.03) | 0.74 (0.56-0.98) |
| **Per allele** |  | **0.89 (0.77-1.02)** | **0.89 (0.77-1.02)** | **0.87 (0.75-1.00)** |
| **rs30099 (5q)** |  |  |  |  |
| CC | 584/1139 | 1.00 | 1.00 | 1.00 |
| CT | 113/248 | 0.89 (0.70-1.13) | 0.89 (0.70-1.14) | 0.90 (0.71-1.16) |
| TT | 11/12 | 1.79 (0.78-4.08) | 1.79 (0.78-4.10) | 1.79 (0.77-4.18) |
| **Per allele** |  | **0.98 (0.79-1.21)** | **0.98 (0.79-1.22)** | **0.99 (0.80-1.24)** |
| **rs4666451 (2p)** |  |  |  |  |
| GG | 272/554 | 1.00 | 1.00 | 1.00 |
| GA | 299/574 | 1.06 (0.87-1.30) | 1.06 (0.87-1.30) | 1.08 (0.88-1.32) |
| AA | 105/204 | 1.05 (0.80-1.38) | 1.05 (0.79-1.38) | 1.06 (0.80-1.40) |
| **Per allele** |  | **1.03 (0.91-1.18)** | **1.03 (0.91-1.18)** | **1.04 (0.91-1.19)** |
| **rs13387042 (2q35)** |  |  |  |  |
| AA | 192/335 | 1.00 | 1.00 | 1.00 |
| AG | 330/657 | 1.08 (0.86-1.36) | 1.08 (0.86-1.35) | 1.08 (0.86-1.37) |
| GG | 163/350 | 1.23 (0.95-1.59) | 1.23 (0.95-1.59) | 1.24 (0.96-1.62) |
| **Per allele** |  | **0.90 (0.79-1.03)** | **0.90 (0.79-1.03)** | **0.90 (0.79-1.02)** |
| **rs7766585 (ESR1)** |  |  |  |  |
| CC | 518/1031 | 1.00 | 1.00 | 1.00 |
| CT | 172/348 | 0.98 (0.80-1.22) | 0.98 (0.80-1.22) | 0.98 (0.79-1.21) |
| TT | 17/26 | 1.30 (0.70-2.42) | 1.30 (0.70-2.42) | 1.42 (0.76-2.67) |
| **Per allele** |  | **1.03 (0.86-1.23)** | **1.03 (0.86-1.23)** | **1.03 (0.86-1.24)** |

* Adjusted for matching variables (age and year of inclusion in study)

** Adjusted for matching variables (age and year of inclusion in study), and for selected confounders (socioeconomic status and exposure to HRT)

| **Rs nr (Gene)** | **p-value**  **inter-action** | **Case/**  **control**  **N** | **Nulliparous**  **OR* (CI 95%)** | **Case/**  **control**  **N** | **Parous**  **OR* (CI 95%)** |
| --- | --- | --- | --- | --- | --- |
| **rs2981582 (FGFR2)** | 0.96 |  |  |  |  |
| CC |  | 30/52 | 1.00 | 200/493 | 1.00 |
| CT |  | 36/69 | 0.90 (0.48-1.69) | 310/571 | 1.36 (1.09-1.69) |
| TT |  | 16/15 | 1.88 (0.78-4.52) | 104/167 | 1.55 (1.15-2.10) |
| **Per allele** |  |  | **1.25 (0.82-1.91)** |  | **1.27 (1.10-1.46)** |
| **rs1045485 (CASP8)** | 0.93 |  |  |  |  |
| CC |  | 21/48 | 1.00 | 162/318 | 1.00 |
| CG |  | 7/14 | 1.08 (0.37-3.18) | 35/71 | 1.03 (0.65-1.63) |
| GG |  | 0/0 | --- | 8/10 | 1.68 (0.64-4.42) |
| **Per allele** |  |  | **0.93 (0.31-2.83)** |  | **1.12 (0.79-1.58)** |
| **rs3803662 (TNRC9)** | 0.58 |  |  |  |  |
| CC |  | 36/72 | 1.00 | 311/694 | 1.00 |
| CT |  | 33/54 | 1.32 (0.71-2.44) | 238/446 | 1.17 (0.95-1.47) |
| TT |  | 8/7 | 1.95 (0.61-6.29) | 54/82 | 1.47 (1.01-2.14) |
| **Per allele** |  |  | **1.33 (0.83-2.14)** |  | **1.19 (1.02-1.40)** |
| **rs8051542 (TNRC9)** | 0.15 |  |  |  |  |
| CC |  | 14/38 | 1.00 | 177/399 | 1.00 |
| CT |  | 41/66 | 1.56 (0.73-3.36) | 289/558 | 1.14 (0.91-1.44) |
| TT |  | 23/28 | 2.18 (0.93-5.14) | 120/234 | 1.14 (0.86-1.52) |
| **Per allele** |  |  | **1.47 (0.96-2.24)** |  | **1.08 (0.94-1.24)** |
| **rs12443621 (TNRC9)** | 0.95 |  |  |  |  |
| AA |  | 22/30 | 1.00 | 170/415 | 1.00 |
| AG |  | 35/79 | 0.68 (0.33-1.42) | 295/565 | 1.28 (1.02-1.62) |
| GG |  | 25/25 | 1.55 (0.67-3.61) | 135/237 | 1.43 (1.08-1.89) |
| **Per allele** |  |  | **1.25 (0.82-1.92)** |  | **1.20 (1.04-1.38)** |
| **rs889312 (MAP3K1)** | 0.11 |  |  |  |  |
| AA |  | 42/67 | 1.00 | 273/653 | 1.00 |
| AC |  | 33/59 | 0.83 (0.46-1.53) | 261/461 | 1.31 (1.06-1.62) |
| CC |  | 4/10 | 0.49 (0.14-1.77) | 61/103 | 1.47 (1.03-2.09) |
| **Per allele** |  |  | **0.77 (0.48-1.24)** |  | **1.24 (1.07-1.45)** |
| **rs3817198 (LSP1)** | 0.48 |  |  |  |  |
| TT |  | 37/68 | 1.00 | 271/591 | 1.00 |
| CT |  | 31/55 | 1.12 (0.60-2.11) | 239/482 | 1.06 (0.85-1.31) |
| CC |  | 8/4 | 4.38 (1.13-16.96) | 68/100 | 1.48 (1.04-207) |
| **Per allele** |  |  | **1.53 (0.93-2.51)** |  | **1.16 (0.99-1.35)** |
| **rs2107425 (H19)** | 0.12 |  |  |  |  |
| CC |  | 44/57 | 1.00 | 311/570 | 1.00 |
| CT |  | 27/57 | 0.54 (0.28-1.05) | 213/498 | 0.80 (0.65-1.00) |
| TT |  | 4/14 | 0.35 (0.11-1.19) | 64/127 | 0.94 (0.67-1.31) |
| **Per allele** |  |  | **0.58 (0.36-0.95)** |  | **0.91 (0.78-1.06)** |
| **rs13281615 (8q24)** | 1.00 |  |  |  |  |
| AA |  | 29/52 | 1.00 | 211/465 | 1.00 |
| AG |  | 37/61 | 1.11 (0.58-2.15) | 290/558 | 1.18 (0.95-1.47) |
| GG |  | 13/19 | 1.32 (0.55-3.15) | 99/183 | 1.26 (0.93-1.70) |
| **Per allele** |  |  | **1.14 (0.75-1.73)** |  | **1.13 (0.98-1.31)** |

**Table 3 Breast cancer risk in relation to selected SNPs with regard to parity**

| **rs981782 (5p12)** | 0.02 |  |  |  |  |
| --- | --- | --- | --- | --- | --- |
| TT |  | 15/38 | 1.00 | 162/290 | 1.00 |
| TG |  | 43/72 | 1.67 (0.79-3.53) | 303/595 | 0.85 (0.67-1.09) |
| GG |  | 14/16 | 2.66 (0.99-7.12) | 107/273 | 0.65 (0.48-0.87) |
| **Per allele** |  |  | **1.64 (1.01-2.67)** |  | **0.81 (0.70-0.94)** |
| **rs30099 (5q)** | 0.47 |  |  |  |  |
| CC |  | 66/101 | 1.00 | 505/1015 | 1.00 |
| CT |  | 14/34 | 0.68 (0.32-1.43) | 97/205 | 0.95 (0.73-1.24) |
| TT |  | 2/1 | 3.18 (0.26-39.46) | 9/11 | 1.63 (0.66-4.06) |
| **Per allele** |  |  | **0.89 (0.47-1.67)** |  | **1.02 (0.81-1.30)** |
| **rs4666451 (2p)** | 0.37 |  |  |  |  |
| GG |  | 26/51 | 1.00 | 237/494 | 1.00 |
| GA |  | 38/54 | 1.39 (0.69-2.59) | 256/502 | 1.08 (0.87-1.34) |
| AA |  | 15/20 | 1.30 (0.54-3.10) | 88/180 | 1.02 (0.75-1.38) |
| **Per allele** |  |  | **1.17 (0.77-1.79)** |  | **1.02 (0.89-1.18)** |
| **rs13387042 (2q35)** | 0.79 |  |  |  |  |
| AA |  | 22/35 | 1.00 | 164/292 | 1.00 |
| AG |  | 40/60 | 1.11 (0.55-2.25) | 287/580 | 0.87 (0.68-1.11) |
| GG |  | 16/36 | 0.69 (0.30-1.61) | 140/308 | 0.80 (0.60-1.06) |
| **Per allele** |  |  | **0.85 (0.56-1.28)** |  | **0.89 (0.78-1.03)** |
| **rs7766585 (ESR1)** | 0.10 |  |  |  |  |
| CC |  | 63/97 | 1.00 | 443/907 | 1.00 |
| CT |  | 18/33 | 0.76 (0.39-1.51) | 150/310 | 0.99 (0.79-1.24) |
| TT |  | 0/6 | ---- | 17/20 | 1.91 (0.98-3.71) |
| **Per allele** |  |  | **0.96 (0.82-1.12)** |  | **1.03 (0.97-1.10)** |

*Adjusted for: age, year of inclusion in study, socioeconomic status and exposure to HRT.

| **Rs nr (Gene)** | **p-value**  **inter-action** | **Case/**  **control**  **N** | **Age < 25 years**  **OR* (CI 95%)** | **Case/**  **control**  **N** | **Age > 25 years**  **OR* (CI 95%)** |
| --- | --- | --- | --- | --- | --- |
| **rs2981582 (FGFR2)** | 0.47 |  |  |  |  |
| CC |  | 110/291 | 1.00 | 90/202 | 1.00 |
| CT |  | 191/352 | 1.49 (1.20-1.99) | 119/219 | 1.18 (0.84-1.66) |
| TT |  | 60/95 | 1.68 (1.13-2.51) | 44/71 | 1.38 (0.87-2.20) |
| **Per allele** |  |  | **1.33 (1.10-1.61)** |  | **1.17 (0.94-1.47)** |
| **rs1045485 (CASP8)** | 0.26 |  |  |  |  |
| CC |  | 104/192 | 1.00 | 58/126 | 1.00 |
| CG |  | 21/41 | 0.99 (0.55-1.80) | 14/30 | 1.14 (0.55-2.34) |
| GG |  | 3/7 | 0.88 (0.22-3.60) | 5/3 | 3.21 (0.71-14.61) |
| **Per allele** |  |  | **0.93 (0.58-1.49)** |  | **1.38 (0.79-2.42)** |
| **rs3803662 (TNRC9)** | 0.91 |  |  |  |  |
| CC |  | 185/419 | 1.00 | 126/275 | 1.00 |
| CT |  | 143/257 | 1.25 (0.95-1.37) | 95/188 | 1.08 (0.78-1.51) |
| TT |  | 31/53 | 1.32 (0.81-2.16) | 23/29 | 1.72 (0.94-3.12) |
| **Per allele** |  |  | **1.19 (0.97-1.46)** |  | **1.21 (0.94-1.55)** |
| **rs8051542 (TNRC9)** | 0.48 |  |  |  |  |
| CC |  | 112/256 | 1.00 | 65/143 | 1.00 |
| CT |  | 166/313 | 1.23 (0.91-1.65) | 123/244 | 1.06 (0.73-1.53) |
| TT |  | 68/149 | 1.01 (0.70-1.46) | 52/85 | 1.36 (0.85-2.16) |
| **Per allele** |  |  | **1.03 (0.86-1.23)** |  | **1.16 (0.92-1.46)** |
| **rs12443621 (TNRC9)** | 0.82 |  |  |  |  |
| AA |  | 104/269 | 1.00 | 66/146 | 1.00 |
| AG |  | 169/312 | 1.48 (1.10-2.01) | 126/252 | 1.07 (0.74-1.55) |
| GG |  | 79/146 | 1.45 (1.01-2.08) | 56/91 | 1.39 (0.88-2.18) |
| **Per allele** |  |  | **1.22 (1.02-1.46)** |  | **1.17 (0.93-1.46)** |
| **rs889312 (MAP3K1)** | 0.65 |  |  |  |  |
| AA |  | 153/371 | 1.00 | 120/282 | 1.00 |
| AC |  | 161/288 | 1.30 (0.99-1.71) | 100/172 | 1.33 (0.95-1.86) |
| CC |  | 37/68 | 1.38 (0.88-2.16) | 24/35 | 1.61 (0.91-2.86) |
| **Per allele** |  |  | **1.21 (1.00-1.48)** |  | **1.30 (1.02-1.65)** |
| **rs3817198 (LSP1)** | 0.17 |  |  |  |  |
| TT |  | 160/359 | 1.00 | 111/232 | 1.00 |
| CT |  | 139/287 | 1.05 (0.79-1.39) | 100/195 | 1.04 (0.74-1.46) |
| CC |  | 44/50 | 2.00 (1.27-3.14) | 24/49 | 1.00 (0.58-1.73) |
| **Per allele** |  |  | **1.27 (1.04-1.55)** |  | **1.02 (0.80-1.30)** |
| **rs2107425 (H19)** | 0.09 |  |  |  |  |
| CC |  | 191/329 | 1.00 | 120/240 | 1.00 |
| CT |  | 120/304 | 0.69 (0.52-0.91) | 93/194 | 0.99 (0.71-1.39) |
| TT |  | 35/78 | 0.78 (0.50-1.22) | 29/49 | 1.19 (0.71-2.00) |
| **Per allele** |  |  | **0.81 (0.66-0.99)** |  | **1.06 (0.83-1.33)** |
| **rs13281615 (8q24)** | 0.80 |  |  |  |  |
| AA |  | 124/290 | 1.00 | 87/175 | 1.00 |
| AG |  | 172/323 | 1.27 (0.95-1.69) | 118/234 | 1.04 (0.74-1.48) |
| GG |  | 54/107 | 1.25 (0.84-1.86) | 45/76 | 1.27 (0.80-2.01) |
| **Per allele** |  |  | **1.15 (0.95-1.83)** |  | **1.11 (0.89-1.39)** |

**Table 4 Breast cancer risk in relation to selected SNPs with regard to age at first child-birth**

| **rs981782 (5p12)** | 0.38 |  |  |  |  |
| --- | --- | --- | --- | --- | --- |
| TT |  | 88/173 | 1.00 | 74/117 | 1.00 |
| TG |  | 185/351 | 0.96 (0.70-1.32) | 118/243 | 0.73 (0.50-1.06) |
| GG |  | 67/169 | 0.71 (0.48-1.05) | 40/104 | 0.60 (0.37-0.96) |
| **Per allele** |  |  | **0.85 (0.70-1.03)** |  | **0.77 (0.61-0.98** |
| **rs30099 (5q)** | 0.31 |  |  |  |  |
| CC |  | 299/621 | 1.00 | 206/393 | 1.00 |
| CT |  | 54/108 | 1.02 (0.71-1.46) | 43/97 | 0.83 (0.55-1.25) |
| TT |  | 6/6 | 2.47 (0.77-7.91) | 3/5 | 0.76 (0.16-3.62) |
| **Per allele** |  |  | **1.14 (0.83-1.56)** |  | **0.85 (0.59-1.23)** |
| **rs4666451 (2p)** | 0.24 |  |  |  |  |
| GG |  | 131/301 | 1.00 | 106/192 | 1.00 |
| GA |  | 159/295 | 1.23 (0.92-1.64) | 97/207 | 0.89 (0.63-1.26) |
| AA |  | 53/109 | 1.11 (0.75-1.65) | 35/71 | 0.90 (0.56-1.45) |
| **Per allele** |  |  | **1.09 (0.91-1.31)** |  | **0.93 (0.74-1.17)** |
| **rs13387042 (2q35)** | 0.89 |  |  |  |  |
| AA |  | 95/168 | 1.00 | 69/124 | 1.00 |
| AG |  | 170/348 | 0.86 (0.63-1.19) | 117/232 | 0.89 (0.61-1.30) |
| GG |  | 84/186 | 0.80 (0.55-1.15) | 56/121 | 0.84 (0.54-1.31) |
| **Per allele** |  |  | **0.89 (0.74-1.07)** |  | **0.92 (0.74-1.14)** |
| **rs7766585 (ESR1)** | 0.60 |  |  |  |  |
| CC |  | 249/525 | 1.00 | 194/382 | 1.00 |
| CT |  | 99/199 | 1.07 (0.80-1.43) | 51/110 | 0.88 (0.60-1.29) |
| TT |  | 11/13 | 2.06 (0.89-4.75) | 6/7 | 1.68 (0.55-5.19) |
| **Per allele** |  |  | **1.07 (0.98-1.18)** |  | **0.98 (0.90-1.08)** |

*Adjusted for: age, year of inclusion in study, socioeconomic status and exposure to HRT.

**Appendix1 Breast cancer risk in relation to selected SNPs stratified on parity**

| **Rs nr (Gene)** | **p-value**  **inter-action** | **Case/**  **control**  **N** | **Nullipara**  **OR* (CI 95%)** | **Case/**  **control**  **N** | **1 para**  **OR (CI 95%)** | **Case/**  **control**  **N** | **2 para**  **OR* (CI 95%)** | **Case/**  **Control**  **N** | **>3 para**  **OR* (CI 95%)** | **p-trend: parity** |
| --- | --- | --- | --- | --- | --- | --- | --- | --- | --- | --- |
| **rs2981582 (FGFR2)** | 0.51 (0.96)^#^ |  |  |  |  |  |  |  |  |  |
| CC |  | 30/52 | 1.00 | 54/117 | 1.00 | 97/235 | 1.00 | 49/141 | 1.00 | 0.06 |
| CT |  | 36/69 | 0.90 (0.48-1.69) | 68/148 | 0.97 (0.62-1.51) | 159/264 | 1.55 (1.13-2.13) | 83/159 | 1.54 (0.99-2.38) | 0.59 |
| TT |  | 16/15 | 1.88 (0.78-4.52) | 19/34 | 1.28 (0.64-2.57) | 60/83 | 1.88 (1.23-2.86) | 25/50 | 1.28 (0.70-2.35) | 0.84 |
| **Per allele** |  |  | **1.25 (0.82-1.91)** |  | **1.07 (0.78-1.47)** |  | **1.39 (1.14-1.71)** |  | **1.20 (0.91-1.60)** |  |
| **rs1045485 (CASP8)** | 0.65 (0.93) |  |  |  |  |  |  |  |  |  |
| CC |  | 21/48 | 1.00 | 32/88 | 1.00 | 94/135 | 1.00 | 36/95 | 1.00 | 0.68 |
| CG |  | 7/14 | 1.08 (0.37-3.18) | 7/13 | 1.83 (0.64-5.21) | 18/37 | 0.72 (0.38-1.36) | 10/21 | 1.30 (0.55-3.10) | 0.89 |
| GG |  | --- | --- | 4/2 | 5.64 (0.88-36.3) | 4/4 | 1.41 (0.34-5.83) | 0/4 | --- | 0.07 |
| **Per allele** |  |  | **0.93 (0.31-2.83)** |  | **2.28 (1.04-4.96)** |  | **0.84 (0.51-1.39)** |  | **0.83 (0.40-1.76)** |  |
| **rs3803662 (TNRC9)** | 0.78 (0.58) |  |  |  |  |  |  |  |  |  |
| CC |  | 36/72 | 1.00 | 72/177 | 1.00 | 159/308 | 1.00 | 80/209 | 1.00 | 0.52 |
| CT |  | 33/54 | 1.32 (0.71-2.44) | 53/98 | 1.34 (0.85-2.11) | 120/230 | 0.96 (0.71-1.30) | 65/118 | 1.52 (1.01-2.30) | 0.74 |
| TT |  | 8/7 | 1.95 (0.61-6.29) | 13/21 | 1.64 (0.76-3.56) | 32/43 | 1.37 (0.83-2.28) | 9/18 | 1.58 (0.66-3.76) | 0.36 |
| **Per allele** |  |  | **1.33 (0.83-2.14)** |  | **1.29 (0.93-1.79)** |  | **1.08 (0.87-1.35)** |  | **1.39 (1.00-1.92)** |  |
| **rs8051542 (TNRC9)** | 0.25 (0.15) |  |  |  |  |  |  |  |  |  |
| CC |  | 14/38 | 1.00 | 30/100 | 1.00 | 97/184 | 1.00 | 50/115 | 1.00 | 0.21 |
| CT |  | 41/66 | 1.56 (0.73-3.36) | 76/131 | 1.88 (1.13-3.14) | 141/270 | 0.97 (0.70-1.35) | 72/157 | 1.01 (0.65-1.59) | 0.15 |
| TT |  | 23/28 | 2.18 (0.93-5.14) | 26/59 | 1.51 (0.79-2.87) | 65/109 | 1.13 (0.75-1.70) | 29/66 | 0.95 (0.54-1.68) | 0.25 |
| **Per allele** |  |  | **1.47 (0.96-2.24)** |  | **1.26 (0.93-1.71)** |  | **1.05 (0.86-1.28)** |  | **0.98 (0.74-1.30)** |  |
| **rs12443621 (TNRC9)** | 0.17 (0.95) |  |  |  |  |  |  |  |  |  |
| AA |  | 22/30 | 1.00 | 31/94 | 1.00 | 94/191 | 1.00 | 45/130 | 1.00 | 0.18 |
| AG |  | 35/79 | 0.68 (0.33-1.42) | 77/139 | 1.59 (0.95-2.67) | 144/279 | 1.06 (0.76-1.47) | 74/147 | 1.62 (1.02-2.55) | 0.85 |
| GG |  | 25/25 | 1.55 (0.67-3.61) | 29/56 | 1.60 (0.85-2.99) | 72/112 | 1.30 (0.88-1.93) | 34/69 | 1.63 (0.94-2.84) | 0.14 |
| **Per allele** |  |  | **1.25 (0.82-1.92)** |  | **1.28 (0.94-1.74)** |  | **1.13 (0.93-1.38)** |  | **1.30 (0.99-1.70)** |  |
| **rs889312 (MAP3K1)** | 0.70 (0.11) |  |  |  |  |  |  |  |  |  |
| AA |  | 42/67 | 1.00 | 63/161 | 1.00 | 140/308 | 1.00 | 70/184 | 1.00 | 0.15 |
| AC |  | 33/59 | 0.83 (0.46-1.53) | 58/111 | 1.25 (0.80-1.97) | 138/221 | 1.37 (1.02-1.85) | 65/129 | 1.20 (0.79-1.83) | 0.90 |
| CC |  | 4/10 | 0.49 (0.14-1.77) | 15/20 | 2.06 (0.96-4.39) | 28/50 | 1.38 (0.83-2.32) | 18/33 | 1.41 (0.73-2.72) | 0.91 |
| **Per allele** |  |  | **0.77 (0.48-1.24)** |  | **1.37 (0.99-1.89)** |  | **1.24 (1.00-1.55)** |  | **1.19 (0.89-1.60)** |  |
| **rs3817198 (LSP1)** | 0.59 (0.48) |  |  |  |  |  |  |  |  |  |
| TT |  | 37/68 | 1.00 | 66/138 | 1.00 | 136/283 | 1.00 | 69/170 | 1.00 | 0.26 |
| CT |  | 31/55 | 1.12 (0.60-2.11) | 49/122 | 0.79 (0.50-1.25) | 129/224 | 1.21 (0.89-1.64) | 61/136 | 1.07 (0.70-1.65) | 0.93 |
| CC |  | 8/4 | 4.38 (1.13-17.0) | 16/25 | 1.35 (0.65-2.78) | 32/49 | 1.29 (0.78-2.14) | 20/26 | 2.00 (1.03-3.89) | 0.49 |
| **Per allele** |  |  | **1.52 (0.93-2.51)** |  | **1.01 (0.73-1.41)** |  | **1.17 (0.94-1.45)** |  | **1.30 (0.96-1.75)** |  |
| **rs2107425 (H19)** | 0.26 (0.12) |  |  |  |  |  |  |  |  |  |
| CC |  | 44/57 | 1.00 | 66/127 | 1.00 | 153/286 | 1.00 | 92/157 | 1.00 | 0.50 |
| CT |  | 27/57 | 0.54 (0.28-1.05) | 52/125 | 0.80 (0.50-1.25) | 114/231 | 0.93 (0.69-1.26) | 47/142 | 0.58 (0.38-0.90) | 0.32 |
| TT |  | 4/14 | 0.35 (0.11-1.19) | 15/34 | 0.83 (0.41-1.66) | 34/51 | 1.23 (0.76-2.01) | 15/42 | 0.59 (0.30-1.13) | 0.86 |
| **Per allele** |  |  | **0.58 (0.36-0.95)** |  | **0.87 (0.63-1.20)** |  | **1.05 (0.84-1.30)** |  | **0.69 (0.52-0.94)** |  |
| **rs13281615 (8q24)** | 0.90 (1.00) |  |  |  |  |  |  |  |  |  |
| AA |  | 29/52 | 1.00 | 51/110 | 1.00 | 103/216 | 1.00 | 57/139 | 1.00 | 0.33 |
| AG |  | 37/61 | 1.11 (0.58-2.15) | 67/131 | 1.14 (0.72-1.81) | 154/281 | 1.21 (0.88-1.66) | 69/146 | 1.23 (0.79-1.90) | 0.44 |
| GG |  | 13/19 | 1.32 (0.55-3.15) | 21/51 | 1.00 (0.53-1.87) | 52/74 | 1.62 (1.05-2.51) | 28/58 | 1.12 (0.63-2.00) | 0.76 |
| **Per allele** |  |  | **1.14 (0.79-1.73)** |  | **1.03 (0.76-1.39)** |  | **1.25 (1.02-1.55)** |  | **1.09 (0.83-1.43)** |  |
| **rs981782 (5p12)** | 0.35 (0.02) |  |  |  |  |  |  |  |  |  |
| TT |  | 15/38 | 1.00 | 35/75 | 1.00 | 84/129 | 1.00 | 43/86 | 1.00 | 0.39 |
| TG |  | 43/72 | 1.67 (0.79-3.53) | 65/139 | 0.99 (0.59-1.65) | 157/286 | 0.83 (0.58-1.17) | 81/170 | 0.87 (0.54-1.39) | 0.60 |
| GG |  | 14/16 | 2.66 (0.99-7.12) | 27/64 | 0.93 (0.50-1.75) | 55/141 | 0.57 (0.37-0.87) | 25/68 | 0.65 (0.35-1.20) | 0.10 |
| **Per allele** |  |  | **1.64 (1.01-2.67)** |  | **0.98 (0.72-1.34)** |  | **0.76 (0.62-0.94)** |  | **0.83 (0.61-1.13)** |  |
| **rs30099 (5q)** | 0.68 (0.47) |  |  |  |  |  |  |  |  |  |
| CC |  | 66/101 | 1.00 | 118/241 | 1.00 | 259/480 | 1.00 | 128/294 | 1.00 | 0.09 |
| CT |  | 14/34 | 0.68 (0.32-1.43) | 20/56 | 0.77 (0.43-1.37) | 52/97 | 1.03 (0.70-1.50) | 25/52 | 1.12 (0.66-1.93) | 0.39 |
| TT |  | 2/1 | 3.18 (0.26-39.5) | 2/1 | 4.15 (0.35-49.0) | 4/7 | 0.91 (0.25-3.29) | 3/3 | 2.42 (0.46-12.7) | 0.49 |
| **Per allele** |  |  | **0.89 (0.47-1.67)** |  | **0.92 (0.54-1.54)** |  | **1.02 (0.73-1.42)** |  | **1.23 (0.78-1.96)** |  |
| **rs4666451 (2p)** | 0.86 (0.37) |  |  |  |  |  |  |  |  |  |
| GG |  | 26/51 | 1.00 | 59/126 | 1.00 | 119/234 | 1.00 | 59/134 | 1.00 | 0.71 |
| GA |  | 38/54 | 1.34 (0.69-2.59) | 57/122 | 1.08 (0.69-1.71) | 128/232 | 1.14 (0.83-1.57) | 71/148 | 1.04 (0.67-1.61) | 0.35 |
| AA |  | 15/20 | 1.30 (0.54-3.10) | 22/38 | 1.38 (0.73-2.60) | 46/84 | 1.03 (0.67-1.58) | 20/58 | 0.79 (0.43-1.45) | 0.07 |
| **Per allele** |  |  | **1.17 (0.77-1.79)** |  | **1.15 (0.85-1.55)** |  | **1.04 (0.85-1.27)** |  | **0.92 (0.69-1.22)** |  |
| **rs13387042 (2q35)** | 0.81 (0.79) |  |  |  |  |  |  |  |  |  |
| AA |  | 22/35 | 1.00 | 39/61 | 1.00 | 85/142 | 1.00 | 40/89 | 1.00 | 0.23 |
| AG |  | 40/60 | 1.59 (0.75-3.38) | 62/149 | 0.89 (0.53-1.48) | 148/264 | 1.11 (0.78-1.60) | 77/167 | 1.13 (0.68-1.86) | 0.53 |
| GG |  | 16/36 | 1.44 (0.62-3.33) | 36/78 | 1.51 (0.84-2.71) | 70/148 | 1.23 (0.82-1.83) | 34/82 | 1.07 (0.61-1.90) | 0.82 |
| **Per allele** |  |  | **0.85 (0.56-1.28)** |  | **0.83 (0.62-1.12)** |  | **0.90 (0.74-1.10)** |  | **0.96 (0.72-1.28)** |  |
| **rs7766585 (ESR1)** | 0.86 (0.10) |  |  |  |  |  |  |  |  |  |
| CC |  | 63/97 | 1.00 | 109/227 | 1.00 | 217/429 | 1.00 | 117/251 | 1.00 | 0.21 |
| CT |  | 18/33 | 0.77 (0.39-1.52) | 26/67 | 0.83 (0.49-1.39) | 88/150 | 1.16 (0.84-1.60) | 36/93 | 0.82 (0.52-1.30) | 0.65 |
| TT |  | 0/6 | --- | 5/5 | 1.81 (0.50-6.56) | 9/11 | 1.81 (0.72-4.54) | 3/4 | 2.42 (0.47-12.5) | 0.19 |
| **Per allele** |  |  | **0.60 (0.33-1.09)** |  | **0.98 (0.64-1.50)** |  | **1.21 (0.92-1.60)** |  | **0.94 (0.62-1.42)** |  |

*Adjusted for: age, year of inclusion in study, socioeconomic status and exposure to HRT.

^#^ Dichotomized; nulliparous versus parous.

**Appendix 2 Breast cancer risk in relation to selected SNPs stratified on age at first child-birth**

| **Rs nr (Gene)** | **p-value**  **inter-action** | **Case/**  **control**  **N** | **<20 years**  **OR* (CI 95%)** | **Case/**  **control**  **N** | **>20- <25 years**  **OR (CI 95%)** | **Case/**  **control**  **N** | **>25- <30 years**  **OR* (CI 95%)** | **Case/**  **Control**  **N** | | **>30 years**  **OR* (CI 95%)** | **p-trend:**  **age at first child-birth** |
| --- | --- | --- | --- | --- | --- | --- | --- | --- | --- | --- | --- |
| **rs2981582 (FGFR2)** | 0.26 (0.47) ^#^ |  |  |  |  |  |  |  |  | |  |
| CC |  | 39/91 | 1.00 | 71/200 | 1.00 | 70/150 | 1.00 | 20/52 | 1.00 | | 0.20 |
| CT |  | 59/102 | 1.41 (0.84-2.37) | 132/250 | 1.57 (1.10-2.23) | 79/162 | 1.01 (0.68-1.51) | 40/57 | 1.82 (0.91-3.61) | | 0.41 |
| TT |  | 15/38 | 0.86 (0.41-1.81) | 45/57 | 2.25 (1.37-3.68) | 30/55 | 1.18 (0.68-2.03) | 14/16 | 2.13 (0.84-5.41) | | 0.57 |
| **Per allele** |  |  | **1.02 (0.73-1.43)** |  | **1.51 (1.19-1.91)** |  | **1.06 (0.82-1.38)** |  | **1.52 (0.97-2.38)** | |  |
| **rs1045485 (CASP8)** | 0.71 (0.26) |  |  |  |  |  |  |  |  | |  |
| CC |  | 34/66 | 1.00 | 70/126 | 1.00 | 44/92 | 1.00 | 14/34 | 1.00 | | 0.38 |
| CG |  | 8/16 | 1.09 (0.41-2.91) | 13/25 | 1.06 (0.50-2.27) | 9/24 | 0.89 (0.37-2.12) | 5/6 | 2.51 (0.62-10.2) | | 0.84 |
| GG |  | 1/4 | 0.62 (0.06-6.00) | 2/3 | 1.18 (0.18-7.62) | 3/2 | 2.19 (0.32-15.2) | 2/1 | 4.53 (0.35-59.3) | | 0.15 |
| **Per allele** |  |  | **0.87 (0.41-1.87)** |  | **1.02 (0.55-1.90)** |  | **1.01 (0.50-2.04)** |  | **4.72 (1.00-22.1)** | |  |
| **rs3803662 (TNRC9)** | 0.76 (0.91) |  |  |  |  |  |  |  |  | |  |
| CC |  | 51/125 | 1.00 | 134/294 | 1.00 | 92/205 | 1.00 | 34/70 | 1.00 | | 0.92 |
| CT |  | 49/86 | 1.34 (0.82-2.21) | 94/171 | 1.22 (0.88-1.71) | 63/144 | 0.94 (0.63-1.39) | 32/44 | 1.38 (0.73-2.63) | | 0.98 |
| TT |  | 9/13 | 1.60 (0.61-4.17) | 22/40 | 1.16 (0.65-2.06) | 18/22 | 1.83 (0.92-3.64) | 5/7 | 1.17 (0.33-4.22) | | 0.38 |
| **Per allele** |  |  | **1.31 (0.89-1.92)** |  | **1.13 (0.89-1.44)** |  | **1.17 (0.87-1.56)** |  | **1.22 (0.74-2.02)** | |  |
| **rs8051542 (TNRC9)** | 0.61 (0.48) |  |  |  |  |  |  |  |  | |  |
| CC |  | 35/91 | 1.00 | 77/165 | 1.00 | 46/108 | 1.00 | 19/35 | 1.00 | | 0.38 |
| CT |  | 50/89 | 1.42 (0.82-2.46) | 116/224 | 1.15 (0.80-1.65) | 84/190 | 1.00 (0.64-1.56) | 39/54 | 1.12 (0.54-2.34) | | 0.87 |
| TT |  | 20/42 | 1.05 (0.82-2.46) | 48/107 | 0.98 (0.62-1.53) | 38/59 | 1.51 (0.87-2.61) | 14/26 | 0.96 (0.38-2.41) | | 0.86 |
| **Per allele** |  |  | **1.07 (0.77-1.49)** |  | **1.00 (0.80-1.24)** |  | **1.21 (0.92-1.60)** |  | **1.00 (0.64-1.58)** | |  |
| **rs12443621 (TNRC9)** | 0.91 (0.82) |  |  |  |  |  |  |  |  | |  |
| AA |  | 26/69 | 1.00 | 78/200 | 1.00 | 45/112 | 1.00 | 21/34 | 1.00 | | 0.84 |
| AG |  | 57/106 | 1.54 (0.86-2.75) | 112/206 | 1.48 (1.04-2.13) | 90/189 | 1.17 (0.75-1.82) | 36/63 | 0.86 (0.41-1.77) | | 0.81 |
| GG |  | 26/49 | 1.45 (0.74-2.87) | 53/97 | 1.42 (0.92-2.20) | 41/66 | 1.59 (0.93-2.72) | 15/25 | 0.96 (0.40-2.32) | | 0.39 |
| **Per allele** |  |  | **1.22 (0.87-1.70)** |  | **1.21 (0.98-1.50)** |  | **1.25 (0.95-1.63)** |  | **0.96 (0.62-1.49)** | |  |
| **rs889312 (MAP3K1)** | 0.49 (0.65) |  |  |  |  |  |  |  |  | |  |
| AA |  | 58/113 | 1.00 | 95/258 | 1.00 | 84/214 | 1.00 | 36/68 | 1.00 | | 0.89 |
| AC |  | 43/89 | 0.95 (0.57-1.58) | 118/199 | 1.54 (1.10-2.15) | 73/132 | 1.42 (0.96-2.10) | 27/40 | 1.20 (0.62-2.34) | | 0.81 |
| CC |  | 8/22 | 0.69 (0.28-1.69) | 29/46 | 1.84 (1.08-3.15) | 15/26 | 1.44 (0.71-2.90) | 9/9 | 2.07 (0.68-6.26) | | 0.34 |
| **Per allele** |  |  | **0.85 (0.59-1.24)** |  | **1.41 (1.11-1.79)** |  | **1.29 (0.97-1.73)** |  | **1.36 (0.84-2.19)** | |  |
| **rs3817198 (LSP1)** | 0.09 (0.17) |  |  |  |  |  |  |  |  | |  |
| TT |  | 48/122 | 1.00 | 112/237 | 1.00 | 85/172 | 1.00 | 26/60 | 1.00 | | 0.61 |
| CT |  | 41/85 | 1.27 (0.75-2.15) | 98/202 | 0.99 (0.70-1.39) | 63/146 | 0.86 (0.57-1.29) | 37/49 | 1.77 (0.89-3.52) | | 0.46 |
| CC |  | 16/11 | 3.71 (1.55-8.89) | 28/39 | 1.59 (0.92-2.76) | 15/39 | 0.79 (0.41-1.53) | 9/10 | 2.84 (0.92-8.77) | | 0.02 |
| **Per allele** |  |  | **1.64 (1.13-2.39)** |  | **1.16 (0.91-1.48)** |  | **0.88 (0.66-1.18)** |  | **1.82 (1.09-3.04)** | |  |
| **rs2107425 (H19)** | 0.04 (0.09) |  |  |  |  |  |  |  |  | |  |
| CC |  | 62/96 | 1.00 | 129/233 | 1.00 | 90/173 | 1.00 | 30/67 | 1.00 | | 0.93 |
| CT |  | 37/93 | 0.60 (0.36-1.00) | 83/211 | 0.73 (0.52-1.02) | 59/153 | 0.75 (0.50-1.13) | 34/41 | 1.86 (0.95-3.64) | | 0.53 |
| TT |  | 8/31 | 0.45 (0.19-1.06) | 27/47 | 1.00 (0.59-1.71) | 23/38 | 1.10 (0.61-1.99) | 6/11 | 1.61 (0.50-5.14) | | 0.57 |
| **Per allele** |  |  | **0.64 (0.44-0.93)** |  | **0.90 (0.70-1.14)** |  | **0.95 (0.72-1.25)** |  | **1.51 (0.93-2.47)** | |  |
| **rs13281615 (8q24)** | 0.84 (0.80) |  |  |  |  |  |  |  |  | |  |
| AA |  | 43/98 | 1.00 | 81/192 | 1.00 | 58/135 | 1.00 | 29/40 | 1.00 | | 0.45 |
| AG |  | 51/96 | 1.35 (0.80-2.25) | 121/227 | 1.30 (0.92-1.85) | 86/174 | 1.16 (0.77-1.76) | 32/60 | 0.71 (0.36-1.42) | | 0.37 |
| GG |  | 13/29 | 1.12 (0.51-2.43) | 41/78 | 1.29 (0.80-2.07) | 33/57 | 1.46 (0.84-2.52) | 12/19 | 0.80 (0.32-2.04) | | 0.08 |
| **Per allele** |  |  | **1.13 (0.79-1.60)** |  | **1.15 (0.92-1.45)** |  | **1.21 (0.93-1.58)** |  | **0.85 (0.54-1.34)** | |  |
| **rs981782 (5p12)** | 0.86 (0.38) |  |  |  |  |  |  |  |  | |  |
| TT |  | 26/52 | 1.00 | 62/121 | 1.00 | 49/82 | 1.00 | 25/35 | 1.00 | | 0.63 |
| TG |  | 55/108 | 0.96 (0.52-1.77) | 130/243 | 0.98 (0.67-1.45) | 89/187 | 0.74 (0.47-1.16) | 29/56 | 0.61 (0.29-1.27) | | 0.29 |
| GG |  | 22/49 | 0.95 (0.46-1.97) | 45/120 | 0.65 (0.40-1.05) | 27/81 | 0.54 (0.30-0.97) | 13/23 | 0.81 (0.32-2.01) | | 0.63 |
| **Per allele** |  |  | **0.97 (0.67-1.40)** |  | **0.82 (0.65-1.03)** |  | **0.74 (0.56-0.99)** |  | **0.85 (0.54-1.34)** | |  |
| **rs30099 (5q)** | 0.53 (0.31) |  |  |  |  |  |  |  |  | |  |
| CC |  | 90/188 | 1.00 | 209/433 | 1.00 | 142/301 | 1.00 | 64/92 | 1.00 | | 0.87 |
| CT |  | 18/35 | 0.98 (0.51-1.86) | 36/73 | 0.97 (0.62-1.52) | 35/69 | 1.05 (0.66-1.68) | 8/28 | 0.45 (0.18-1.07) | | 0.35 |
| TT |  | 2/3 | 1.34 (0.20-8.97) | 4/3 | 3.48 (0.72-16.8) | 2/3 | 0.98 (0.13-7.37) | 1/2 | 0.68 (0.04-11.4) | | 0.50 |
| **Per allele** |  |  | **1.04 (0.60-1.80)** |  | **1.13 (0.76-1.68)** |  | **1.05 (0.68-1.62)** |  | **0.52 (0.24-1.10)** | |  |
| **rs4666451 (2p)** | 0.34 (0.24) |  |  |  |  |  |  |  |  | |  |
| GG |  | 41/90 | 1.00 | 90/211 | 1.00 | 74/149 | 1.00 | 32/43 | 1.00 | | 0.18 |
| GA |  | 54/95 | 1.26 (0.74-2.14) | 105/200 | 1.04 (0.70-1.54) | 64/153 | 1.20 (0.75-1.93) | 33/54 | 0.71 (0.32-1.55) | | 0.20 |
| AA |  | 13/37 | 0.79 (0.36-1.71) | 40/72 | 1.06 (0.68-1.65) | 29754 | 1.19 (0.70-2.01) | 6/17 | 1.09 (0.45-2.62) | | 0.97 |
| **Per allele** |  |  | **0.97 (0.69-1.38)** |  | **1.13 (0.91-1.42)** |  | **1.01 (0.77-1.31)** |  | **0.67 (0.42-1.09)** | |  |
| **rs13387042 (2q35)** | 0.83 (0.89) |  |  |  |  |  |  |  |  | |  |
| AA |  | 32/46 | 1.00 | 63/122 | 1.00 | 47/97 | 1.00 | 22/27 | 1.00 | | 0.62 |
| AG |  | 54/116 | 1.15 (0.62-2.12) | 116/232 | 0.98 (0.66-1.44) | 84/170 | 1.01 (0.65-1.58) | 33/62 | 0.65 (0.31-1.37) | | 0.09 |
| GG |  | 23/55 | 1.64 (0.81-3.31) | 61/131 | 0.94 (0.60-1.46) | 38/93 | 0.85 (0.50-1.43) | 18/28 | 0.92 (0.38-2.21) | | 0.15 |
| **Per allele** |  |  | **0.78 (0.55-1.11)** |  | **0.96 (0.77-1.20)** |  | **0.92 (0.71-1.19)** |  | **0.95 (0.61-1.49)** | |  |
| **rs7766585 (ESR1)** | 0.92 (0.60) |  |  |  |  |  |  |  |  | |  |
| CC |  | 69/160 | 1.00 | 180/365 | 1.00 | 139/288 | 1.00 | 55/94 | 1.00 | | 0.72 |
| CT |  | 35/63 | 1.45 (0.85-2.45) | 64/136 | 0.96 (0.67-1.37) | 35/83 | 0.82 (0.52-1.30) | 16/27 | 0.86 (0.41-1.81) | | 0.64 |
| TT |  | 5/5 | 2.74 (0.72-10.44) | 6/8 | 1.61 (0.54-4.86) | 5/5 | 2.13 (0.59-7.67) | 1/2 | 0.40 (0.03-5.22) | | 0.24 |
| **Per allele** |  |  | **1.51 (0.97-2.36)** |  | **1.03 (0.75-1.41)** |  | **0.98 (0.66-1.44)** |  | **0.79 (0.40-1.45)** | |  |

*Adjusted for: age, year of inclusion in study, socioeconomic status and exposure to HRT.

^#^ Dichotomized; age at first childbirth < 25 years of age versus age at first childbirth > 25 years of age.
